# Impact of M-POSS on Selected Properties of Experimental Methacrylate Matrices and Composites †

**DOI:** 10.3390/ma19061261

**Published:** 2026-03-23

**Authors:** Kinga Bociong, Barbara Kosior, Norbert Soboń, Monika Domarecka, Jerzy Sokołowski, Aleksandra Zimon, Michał Krasowski, Agata Szczesio-Wlodarczyk

**Affiliations:** 1 Department of General Dentistry, Medical University of Lodz, 251 Pomorska St., 92-213 Lodz, Poland; 2 Institute of General and Ecological Chemistry, Faculty of Chemistry, Lodz University of Technology, Zeromskiego 114, 90-543 Lodz, Poland; aleksandra.zimon@p.lodz.pl; 3 University Laboratory of Materials Research, Medical University of Lodz, Pomorska 251, 92-213 Lodz, Poland

**Keywords:** polymer matrix, resin dental composite, modification, polyhedral oligomeric silsequioxanes, mechanical properties, contraction stress

## Abstract

Methacrylate-POSS (M-POSS) is a novel organic–inorganic additive shown to reinforce dental composites and reduce polymerization shrinkage. This study aimed to evaluate the influence of M-POSS addition (0.5, 2, 10, or 15 wt.%) on the mechanical properties of an experimental polymer matrix (bis-GMA/UDMA/TEGDMA/HEMA = 35/35/20/10 wt.%) and a dental resin composite (45 wt.% silanized silica as filler). Vickers hardness (HV), three-point bending strength (FS), diametral tensile strength (DTS), and shrinkage stress generated during polymerization were studied. The results show HV values between 16 and 18 compared to 15 ± 1 in the control group. Hardness in the control composite was 34 ± 4, and after modification, it showed similar or slightly lower values between 32 and 35. FS increased from 90 ± 4 MPa before modification to 100 ± 5 MPa for 2 wt.% M-POSS, and then decreased to 78 ± 5 MPa for materials containing 15 wt.% M-POSS. FS of composites were within the range of 61–77 MPa, with a similar tendency in variation to that of matrices. DTS values decreased after M-POSS addition, from 37 ± 4 MPa before modification to 31–33 MPa after modification. Flexural modulus decreases after modification, both for matrices and composites. The morphology of composites with >10 wt. % M-POSS showed visible surface irregularities. In conclusion, M-POSS affects matrix hardness, resulting in an increase in HV. The addition of M-POSS also increases FS values of the matrix, but only up to a certain concentration. However, the introduction of M-POSS does not significantly affect the HV or bending strength of the composites. Although DTS values decreased, this change was not statistically significant. Finally, contraction stress was significantly reduced for groups containing 2 wt.% and 10 wt.% M-POSS, representing an anticipated and promising improvement.

## 1. Introduction

Resin-based dental composites (RDCs) appear to be the most favoured material for replacing tooth tissue today [1,2]. The fundamentals of minimally invasive dentistry are based on restorative treatment and the ability to maximally preserve tooth structure, which is possible due to enhanced properties of modern RDCs. Dental composites, unlike previous amalgam fillings or more invasive fixed prosthetic restorations, allow for good adhesion to enamel and dentine, making it useful for a range of applications [3,4]. Nevertheless, despite their widespread use, this material still exhibits numerous drawbacks. These drawbacks may include polymerization shrinkage, increased wear over time, water sorption, and mechanical failure. These factors can lead to secondary caries, debonding, fracture, and overall restoration failure [5]. To address these concerns, researchers and manufacturers add various compounds to modify specific properties of the composite. RDCs usually consist of two phases: the matrix phase and the filler phase. The matrix, as the organic phase, is composed of various polymers, such as bisphenol A-glycidyl dimethacrylate (Bis-GMA), urethane dimethacrylate (UDMA), triethylene glycol dimethacrylate (TEGDMA), and many other experimental resins, as well as photoinitiators, polymerization inhibitors, and stabilizers [6]. The prepared matrix is then usually filled with silanized silica. It has been established that the properties are affected not only by the amount of filler but also the filling method—whether performed manually or using special mixers [7]. All the mentioned factors provide endless possibilities for improving RDC properties by addressing specific clinical needs.

Polyhedral oligomeric silsesquioxanes (POSS) have recently garnered increased interest in the literature as an additive responsible for various enhancements of RDCs. This compound constitutes a silicon–oxygen cage with a range of organic groups covalently bonded to the core [8]. The substituents can be hydrogen atoms, alkyl, aryl, hydrocarbons, hydroxyl, or amino groups [9]. POSS addition acts as a modifier to both the matrix and filler phase, as it exhibits both organic and inorganic properties [10]. In dentistry, methacrylate-POSS (M-POSS) is currently the most studied compound. It has been shown to enhance the mechanical properties of experimental composites, as well as reduce polymerization shrinkage and water sorption. It acts as a multifunctional crosslinker, making the polymer network more rigid [10,11,12,13]. However, these effects occur only up to a certain addition level. This is because concentrations exceeding a specific threshold may diminish the overall RDC quality due to POSS agglomeration [14]. This specific threshold is difficult to establish, as current research presents diverse methodologies, making comparison challenging. Another important factor is the form of M-POSS provided by the manufacturer. M-POSS in nanosilica dispersion has recently been studied, which again revealed slightly different results, characterized by reduced polymerization shrinkage but less visible improvement in certain mechanical qualities [15].

Dental resin composites modified with M-POSS demonstrate promising results relevant to specific causes of restoration failure, such as polymerization shrinkage and debonding. Nevertheless, due to the non-cohesive nature of the current literature, much research is still required, such as for the commercial implementation of M-POSS. The aim of this article is to test and highlight specific mechanical properties that are enhanced using various concentrations of this novel additive and to further consolidate available knowledge [16]. The first null hypothesis is that M-POSS addition has no significant effect on the properties of the experimental resin matrix. The second null hypothesis is that M-POSS addition does not significantly influence the properties of the experimental composites.

## 2. Materials and Methods

A polymer matrix was prepared with bisphenol A-glycidyl dimethacrylate (bis-GMA, purity ≥ 97%), urethane dimethacrylate (UDMA, purity ≥ 97%), triethylene glycol dimethacrylate (TEGDMA, purity ≥ 95%), and 2-hydroxyethyl methacrylate (HEMA, purity ≥ 97%) (all from Sigma-Aldrich, St. Louis, MO, USA) at a ratio of 35/35/20/10 wt.%. The resin matrix was prepared by weighing bis-GMA, UDMA, TEGDMA, and HEMA into a light-proof glass container. The monomer mixture was pre-mixed manually at 60 °C. Next, the photoinitiator system—comprising 2-(dimethylamine)ethyl methacrylate (DMAEMA, purity ≥ 98%, 0.9 wt.%), butylated hydroxytoluene (BHT, purity ≥ 99%, 0.1 wt.%), and camphorquinone (CQ, purity ≥ 97%, 0.4 wt.%)—was incorporated under darkroom conditions to prevent premature polymerization. Thorough homogenization was achieved via magnetic stirring. The resin matrix was allowed to stabilize for 7 days prior to composite preparation. The mixture was divided evenly into five parts to enable further modification. Then, the methacryloxypropyl polyhedral oligomeric silsesquioxane (octamethacryloxypropyloocta silsesquioxane, M-POSS) modifier was added to each part, respectively, in amounts of 0.5, 2, 10, or 15% by weight. The unmodified matrix was the control. M-POSS is a hybrid molecule with an inorganic silsesquioxane at the core and organic methacrylate groups attached at the corners of the cage, characterized by a weight amount of 1433.97 g/mol and viscosity in the range of 1.5–2.2 Pa·s (25 °C); it was obtained from Hybrid Plastics, Inc. (Hattiesburg, MS, USA). Finally, silanized silica filler Arsil (Zakłady Chemiczne Rudniki S.A., Rudniki, Poland) silanized by 3-methacryloxypropyltri-methoxysilane (γ-MPTS, Unisil Sp.z o.o., Tarnów, Poland) [17] in the amount of 45 wt.% was incorporated using an agate mortar. The silica was added in small portions and manually ground until a homogeneous paste was obtained. The samples of matrices and composites were made using silicone moulds. On each sample surface, a tape (Hawe Striproll, Kerr, Bioggio, Switzerland) and laboratory slides were placed to prevent the formation of an oxygen inhibition layer. Then, the materials (unfilled and filled) were cured for 20 s using a CURE TC-01 polymerisation lamp (SPRING; Norristown, PA, USA) with a power of 1200 mW/cm^2^ at a 1.5 mm material thickness.

The Vickers hardness and flexural strength of the matrices were measured to investigate the effect of M-POSS addition. For composites, the Vickers hardness, flexural strength, diametral tensile strength, and shrinkage stress generated during the photopolymerization were evaluated. Vickers hardness (HV) was determined using a ZHμ-2 semi-automatic hardness tester (Zwick/Roell, Ulm, Germany), with a load of approximately 10 N. Nine measurements were taken for each material. The flexural strength (FS) of the unfilled and filled materials was determined using a three-point bending test (TPB) according to ISO 4049 [15,18], with six specimens measuring 25 × 2 × 2 mm. The traverse speed was 1 mm/min. For diametral tensile strength (DTS), nine specimens were fabricated for each test group, in the form of a cylinder with a height of 3 mm and a diameter of 6 mm, and they were subjected to compression along the diameter, perpendicular to the major axis, until failure. The test was performed with the crosshead moving at 2 mm/min. An elasticoptic method using a Gunt FL200 circular polariscope (Gunt Gerätebau GmbH, Barsbüttel, Germany) was used to evaluate polymerization shrinkage stress generated during the light curing of the composites. Stress was calculated using transformed Timoshenko formulas and elastic theory formulas. An in depth explanation of the method can be found in our earlier work [19,20]. To fully characterize the shrinkage, three stress parameters were analyzed: radial stress (σr), circumferential stress (σθ), and reduced stress (σint). Among these, σint was defined as the primary outcome measure—shrinkage stress. It integrates the multi-axial stress components into a single value that represents the overall stress intensity at the resin–photoelastic interface, which is similar to the stress state on the bonding surface between tissue and replacement materials under clinical conditions.

The surface morphology of the samples was studied using scanning electron microscopy (SEM, Hitachi S-4700, Tokyo, Japan) equipped with Energy-Dispersive X-ray Spectroscopy (EDS, Thermo Scientific UltraDry, Waltham, MA, USA). Prior to testing, the specimens were polished with paper of decreasing gradation to obtain a smooth surface and then sputter-coated with gold (208HR sputter coater, Cressington Scientific Instruments Ltd., Watford, UK). SEM imaging was performed at an accelerating voltage of 25 kV.

Statistical analyses (Statistica v.13, Tibco Software Inc., Krakow, Poland) were performed on all datasets. The normality of the data distribution was first assessed using Shapiro–Wilk tests. When the results indicated an approximate normal distribution, homogeneity of variances was subsequently verified using Levene’s test. For parametric data, statistical comparisons were conducted using one-way analysis of variance (ANOVA), followed by Tukey’s honestly significant difference (HSD) post hoc test. When the assumptions of the parametric analysis were not met, the nonparametric Kruskal–Wallis test was applied, followed by multiple comparisons of mean ranks across groups. A significance level of *p* < 0.05 was adopted for all statistical analyses.

## 3. Results

**Figure A1:**
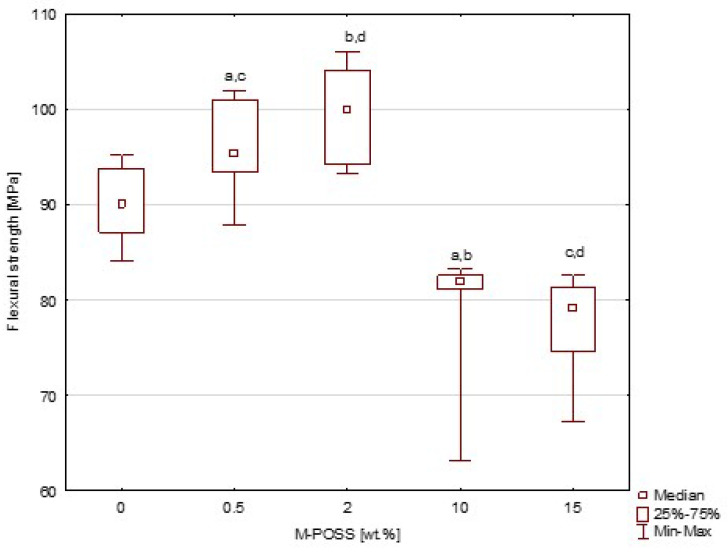
Box-and-whisker plot of flexural strength of tested matrices modified with M-POSS. Statistically significant differences (the same letter) were found between the following: (a,b) 10 wt.% M-POSS and (a) 0.5 wt.% M-POSS (*p*-value = 0.015298) and (b) 2 wt.% M-POSS (*p*-value = 0.003034); (c,d) 15 wt.% M-POSS and (c) 0.5 wt.% M-POSS (*p*-value = 0.001640) and (d) 2 wt.% M-POSS (*p*-value = 0.000253).

**Figure A2:**
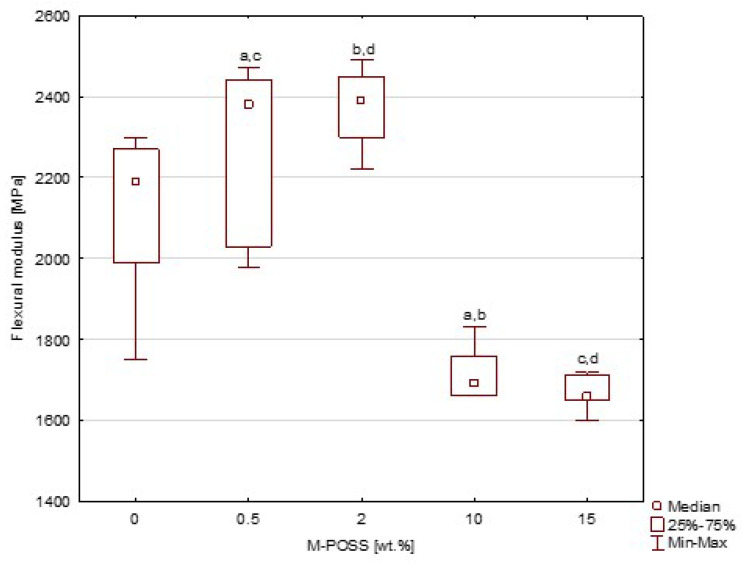
Box-and-whisker plot of flexural modulus of tested matrices modified with M-POSS. Statistically significant differences (the same letter) were found between the following: 0 wt.% M-POSS and (b,d) 2 wt.% M-POSS (*p*-value = 0.02313), (a,b) 10 wt.% M-POSS (*p*-value = 0.00026), and 15 wt.% M-POSS (*p*-value = 0.000054); (a,c) 0.5 wt.% M-POSS and 15 wt.% M-POSS (*p*-value = 0.00000014) and (a,b) 10 wt.% M-POSS (*p*-value = 0.00000062); (b,d) 2 wt.% M-POSS and 10 wt.% M-POSS (*p*-value = 0.000000027) and (c,d) 15 wt.% M-POSS (*p*-value = 0.0000000066).

**Figure A3:**
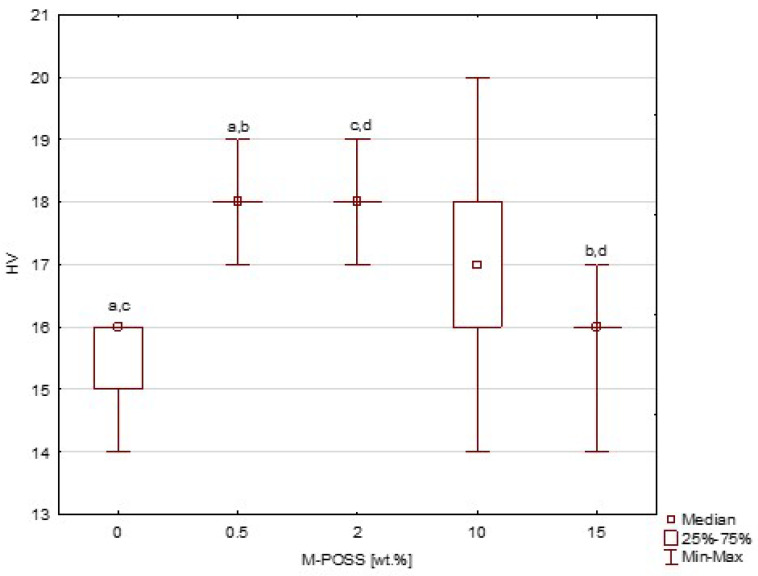
Box-and-whisker plot of Vickers hardness (HV) of tested matrices modified with M-POSS. Statistically significant differences (the same letter) were found between the following: (a,b) 0.5 wt.% M-POSSS and (a) 0 wt.% M-POSS (*p*-value = 0.000678) and (b) 15 wt.% M-POSS (*p*-value = 0.007910); (c,d) 2 wt.% M-POSSS and (c) 0 wt.% M-POSS (*p*-value = 0.002694) and (d) 15 wt.% M-POSS (*p*-value = 0.025703).

The values are presented as the mean with the standard deviation for variables with a normal distribution and homogeneity of variance, or the median with the quartile deviation for non-normally distributed variables. The results obtained for neat resin modified with M-POSS are presented in Table 1 and Figure A1, Figure A2 and Figure A3.

The highest values of flexural strength (99.9 MPa) and flexural modulus (2390 MPa) were exhibited by the matrix modified with 2 wt.% of M-POSS. The lowest values of FS (79.2 MPa) and FM (1660 MPa) were obtained for the material containing 15 wt.% of M-POSS. The Vickers hardness of neat resin modified with M-POSS was in the range of 16–18.

**Figure A4:**
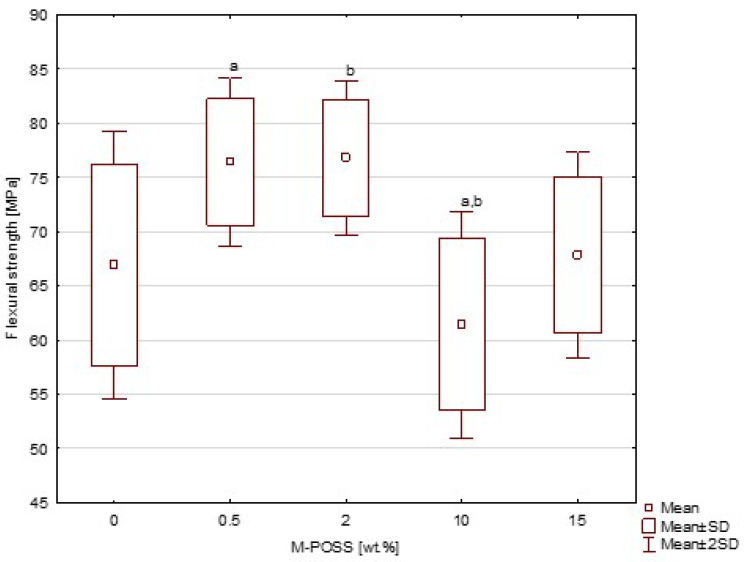
Box-and-whisker plot of flexural strength of tested composites with M-POSS. Statistically significant differences (the same letter) were found between the following: (a,b) 10 wt.% M-POSS and (a) 0.5 wt.% M-POSS (*p*-value = 0.049171) and (b) 2 wt.% M-POSS (*p*-value = 0.041733).

**Figure A5:**
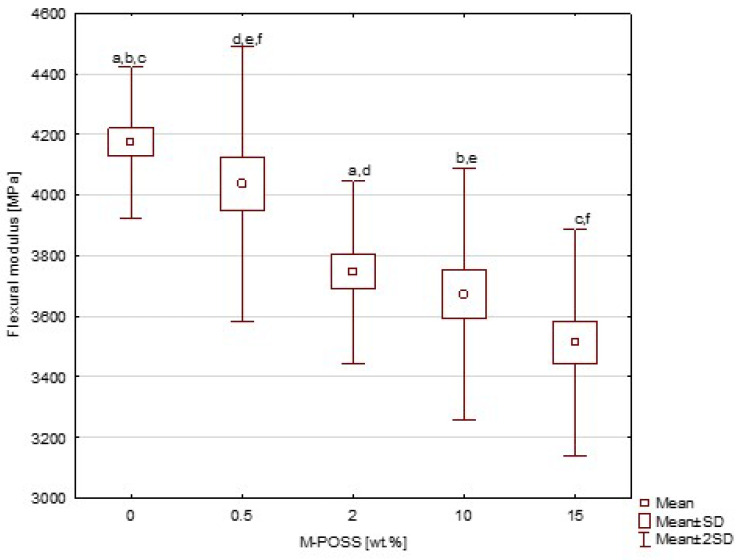
Box-and-whisker plot of flexural modulus of tested composites with M-POSS. Statistically significant differences (the same letter) were found between the following: (a,b,c) 0 wt.% M-POSSS and (a) 2 wt.% M-POSS (*p*-value = 0.001329), (b) 10 wt.% M-POSS (*p*-value = 0.000288), and (c) 10 wt.% M-POSS (*p*-value = 0.000135); (d,e,f) 0.5 wt.% M-POSSS and (d) 2 wt.% M-POSS (*p*-value = 0.042750), (e) 10 wt.% M-POSS (*p*-value = 0.007043), and (f) 10 wt.% M-POSS (*p*-value = 0.000216).

**Figure A6:**
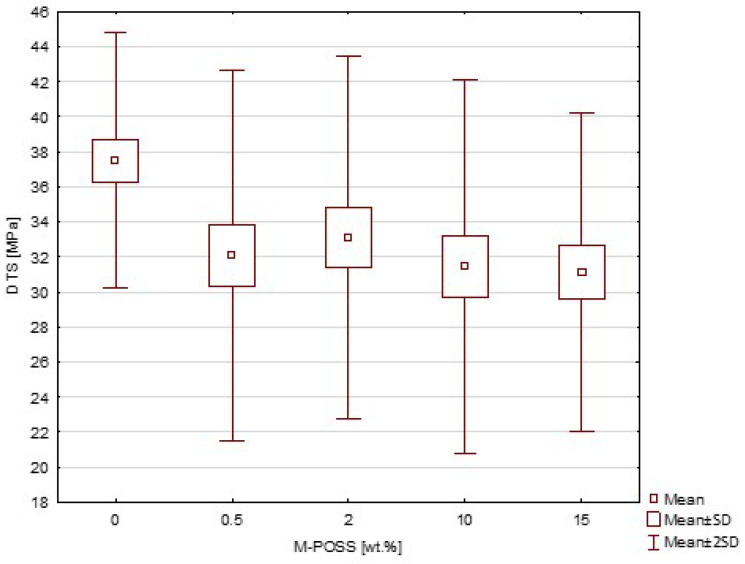
Box-and-whisker plot of diametral tensile strength (DTS) of tested composites with M-POSS. No statistically significant differences were found.

The obtained results (FS, FM, DTS, HV) of the composites with M-POSS are presented in Table 2 and Table S1, Figure A4, Figure A5 and Figure A6.

The highest FS (76.8 MPa) was shown by the composite with 2 wt.% M-POSS. The control group (without M-POSS) exhibited the highest FM (4174 MPa) and DTS (37.5 MPa). The lowest FS, FM, and DTS were obtained by composites with 10 and 15 wt.% M-POSS. The Vickers hardness of composites with M-POSS was in the range of 32–35.

The shrinkage stress results are summarized in Table 3. The isochromes representing reduced stress are shown in Table A1. The table compares the radial stress (σr), circumferential stress (σθ), and reduced stress (σint) generated during the irradiation of the experimental composites with varying M-POSS content (wt.%). The reduced stress (σint) is treated as the main outcome parameter describing the effective polymerization shrinkage stress in the material, while σr and σθ represent the directional components of the stress state and are provided to illustrate the stress distribution within the system.

The reduced stress (σint), which represents the stress state on the border between material and tooth tissue (which is similarly observed with the epoxy resin plate under elastooptic analysis), varied between 15 and almost 19 MPa depending on composition. The lowest stress was observed for composites with 2 or 10 wt.% of M-POSS.

**Figure 1:**
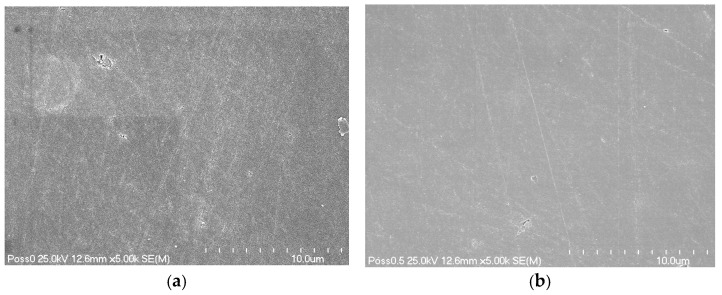

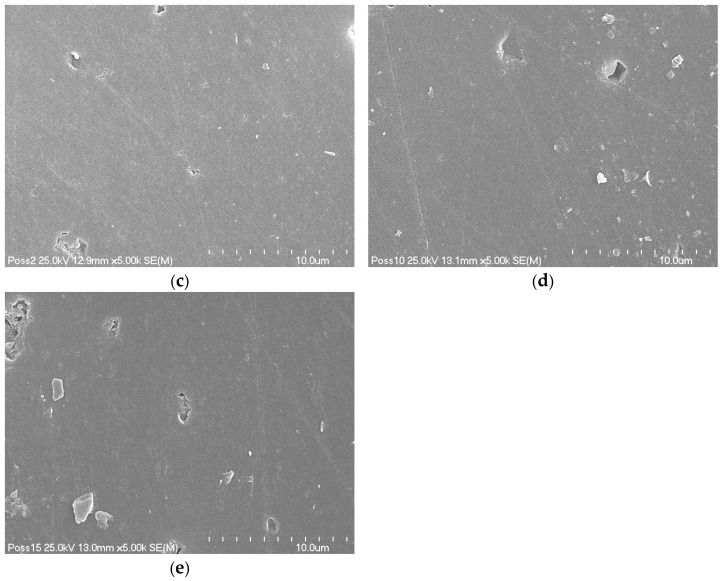
SEM micrographs with magnification 5000× of composite without (**a**) and with M-POSS ((**b**)—0.5 wt.%; (**c**)—2 wt.%; (**d**)—10 wt.%; (**e**)—15 wt.%).

Representative SEM images are presented in Figure 1a–e.

## 4. Discussion

The modification of resin-based dental composites remains a major research focus in contemporary dental materials science. The clinical performance of restorative materials used to reconstruct lost hard dental tissues remains suboptimal [21]. The incorporation of modifiers, including polyhedral oligomeric silsesquioxanes (POSS), into methacrylate-based dental resins is one of the solutions being investigated. Due to their hybrid organic–inorganic structure, POSS derivatives can participate in polymer network formation while simultaneously acting as reinforcing elements [9]. Methacrylate-functionalized POSS (M-POSS) has been shown to chemically integrate into the polymer matrix, potentially influencing the mechanical properties, crosslink density, and stress development during photopolymerization [22]. The influence of M-POSS was initially evaluated in the polymer matrix alone, and subsequently in a composite system containing 45 wt.% silica filler. This experimental design enabled a comprehensive assessment of the modifier’s effect in both isolated and complex composite structures.

After careful analysis of the obtained results, both hypotheses were rejected. Our findings indicate that low concentrations of M-POSS often yield the most favourable balance of properties, which is consistent with other studies [12,13,22,23,24]. Due to its unique hybrid organic–inorganic structure and multiple methacrylate functional groups, M-POSS has significant potential as a reactive modifier within dental resin matrices. The presence of methacrylate groups enables M-POSS to participate in the free-radical polymerization process, forming covalent bonds with surrounding methacrylate monomers and integrating into the polymer network. This integration may lead to a more densely crosslinked structure, which improves load transfer and enhances the mechanical properties and elasticity of the cured material [25,26].

The reinforcing effect of M-POSS depends heavily on its concentration in the resin or composite. At higher loads, generally above 10 wt.%, phase separation, aggregation, or disruption of the polymer network can occur. This leads to uneven regions with weaker structural integrity. Previous studies have linked this to decreased mechanical performance in dental resins containing high levels of POSS, in which clusters can concentrate stress and reduce the material’s strength [15,27,28]. Another possible reason could be that the composite is more brittle due to a higher inorganic phase and/or over-crosslinked when some limit of POSS is reached [12]. Our findings support these observations (Table 2 and Table 3). In both the polymer matrix and the silica-filled composites, the mechanical properties, including flexural strength, elastic modulus, and other parameters, were generally better for materials with low M-POSS additions (0.5 wt.% and 2 wt.%) compared to those with higher concentrations (10 wt.% and 15 wt.%).

Scanning electron microscopy (SEM) provided greater clarity on the microstructural effects associated with increasing M-POSS content. Composites with higher amounts of the modifier showed visible surface irregularities. They also had more features that appeared to be agglomerates or artefacts (Figure 1d,e). This supports the idea of phase separation at higher concentrations. Areas with poor dispersion or clustering of M-POSS might disrupt the local polymer network, leading to localized stress concentrations and a general decrease in bulk mechanical properties.

From our studies, it follows that the optimum amount of M-POSS in dental composites is 2 wt.%. Beyond this threshold, overall mechanical properties are better than the control’s. As stated above, this phenomenon probably results from good dispersion of M-POSS in the resin-based matrix, increased distance between POSS molecules, and, thus, a lower tendency to aggregate. SEM photos confirm this, where materials containing up to 2 wt.% are characterized by a homogeneous structure, without visible aggregates or agglomerates (Figure 1a–c). Additionally multi-methacrylate POSS structures participate in reinforcing tri-dimensional network formation. The incorporation of 10 wt.% or more of M-POSS influences the morphology—more aggregates are visible, and as a result, the mechanical properties decreased.

In terms of overall mechanical performance, resin matrices modified with low concentrations of M-POSS exhibit properties superior to those of dimethacrylate-based resins used in dental research. Unfilled UDMA-based resins typically exhibit flexural strength values of approximately 70–90 MPa and Vickers hardness values around 14–16, depending on the co-monomer composition [29]. The experimental composites developed in this study were close to meeting the 80 MPa threshold required for Type 1 materials (restorations involving occlusal surfaces) and complied with ISO 4049 for Type 2 materials (FS > 50 MPa) [18]. The DTS of our experimental materials ranged from 31 to 38 MPa, values comparable to those of other experimental dental composites and some restorative materials [30,31], but they were lower than those of high-performance commercial composites, which may have DTS values ranging from over 40 to over 60 MPa [32]. In our experiments, the composites showed hardness values just above 30 HV. Although these values are relatively low compared to those of many commercial universal restorative composites, this is mainly due to the lower filler content of 45 wt.% in our formulations. Still, HV remains within the range for some flowable-type materials [33]. Such materials are clinically valued for their lower viscosity and better adaptation to cavity walls. In this context, the developed experimental composites could be considered potential candidates for liner materials. Filler content is a known factor in determining microhardness in resin composites. Higher filler concentrations reinforce the resin matrix and reduce the volume of the polymer network. Commercial composites frequently contain more than 60 wt.% inorganic filler, incorporating various hybrid and nanofillers. Consequently, these materials exhibit significantly higher hardness, enhanced flexural strength, and improved wear resistance [34,35,36]. Therefore, it can be assumed that, with appropriate selection of filler type and loading, the developed experimental composite material could achieve improved mechanical performance. However, the optimization of filler composition was not the objective of the present manuscript. The silica content used in this study was selected to ensure a homogeneous composite structure under laboratory mixing conditions. Moreover, the use of a simple and uniform filler system minimized additional variables that could influence the results, allowing a clearer assessment of the effects associated with resin matrix modification.

The incorporation of M-POSS into resin-based systems aims to reduce polymerization shrinkage and the related shrinkage stress. These issues are still challenging for dental composite materials. In this study, the lowest shrinkage stress results were observed in formulations containing 2 wt.% and 10 wt.% M-POSS. The reduction in shrinkage stress observed in our studies is influenced by several factors. Firstly, in the rigid inorganic cage structure, which undergoes minimal volumetric change during curing, POSS molecules exhibit lower polymerization shrinkage than conventional dimethacrylate monomers [23,37,38]. Secondly, the large free volume associated with POSS can also modify the polymer network, potentially partially compensating for volumetric contraction [27,39,40]. In addition, incorporating POSS molecules into the polymer network may also induce a plasticizing effect, as evidenced by reduced glass transition temperatures reported for POSS-modified systems [27,41]. This may enhance molecular mobility, additionally contributing to the relaxation of developing shrinkage stresses. However, we did not measure glass transition temperature changes in our study, but we observed that composites with low shrinkage stress have a low elastic modulus, which may indicate, with high probability, some plasticization of the material. However, at higher M-POSS contents, these beneficial effects may be counterbalanced by increased network stiffness. The rigid POSS cages can restrict polymer chain mobility and limit stress relaxation, resulting in no further reduction—or even an increase—in shrinkage stress despite lower volumetric shrinkage. Another factor that determines the increase in contraction stress generated by the composite with 15 wt.% is the degree of conversion (DC). In our earlier work [15], a high addition of methacrylate-functionalized POSS dispersed in nanosilica (MA/Ns-POSS) into composites resulted in increased degree of conversion (DC composite with 15 wt.% MA/Ns-POSS reached 78%, while DC composite with 1 wt.% MA/Ns-POSS reached 53%). Furthermore, the effectiveness of M-POSS is determined by its miscibility with resin co-monomers and its homogeneous dispersion within the organic phase. Insufficient compatibility or aggregation at elevated concentrations can reduce the modifier’s stress-reducing capacity.

Overall, the results indicate that achieving optimal mechanical reinforcement in POSS-modified dental materials requires careful control of the additive concentration. Low levels of M-POSS improve mechanical performance and reduce shrinkage stress. In contrast, excessive amounts promote microstructural imperfections, resulting in material weakening. This concentration-dependent behaviour should be considered when designing next-generation dental composites.

## 5. Conclusions

- Regarding the influence of M-POSS addition on the mechanical properties of cured matrices and composites, low concentrations of M-POSS (0.5–2 wt.%) effectively improve the mechanical performance of Bis-GMA/UDMA/TEGDMA/HEMA-based polymer matrices and their corresponding composites.
- A reduction in polymerization shrinkage stress was observed for composites containing only 2 wt.% or 10 wt.% M-POSS, indicating a concentration-dependent effect of the modifier.
- Further enhancement of composite performance may be achieved by increasing filler content and optimizing filler composition; however, maintaining adequate homogeneity under laboratory conditions continues to be a significant challenge.

## Figures and Tables

**Table 1:** The flexural strength (FS), flexural modulus (FM), and hardness (HV) of tested matrices modified with M-POSS. The same letter indicates a statistical difference in the results at the *p* ≤ 0.05 level.

M-POSS Content [wt.%]	Median FS (MPa)	QT	Median FM (MPa)	QT	Median HV	QT
0	90.1	6.7	2190	280	16 ^a,c^	1
0.5	95.3 ^a,b^	7.6	2380 ^a,b^	410	18 ^a,b^	0
2	99.9 ^c,d^	9.7	2390 ^c,d^	150	18 ^c,d^	0
10	81.9 ^a,c^	1.5	1690 ^a,c^	100	17	2
15	79.2 ^b,d^	6.6	1660 ^b,d^	60	16 ^b,d^	0

**Table 2:** The flexural strength (FS), flexural modulus (FM), diametral tensile strength (DTS), and hardness (HV) of composites with M-POSS. The same letter indicates a statistical difference in the results at the *p* ≤ 0.05 level.

M-POSS Content [wt.%]	Mean FS (MPa)	SD	Mean FM (MPa)	SD	Median HV	QT	Mean DTS (MPa)	SD
0	66.9	12.3	4174 ^a–c^	125	34	1	37.5	3.6
0.5	76.4 ^a^	7.7	4037 ^d–f^	227	34	1	32.1	5.3
2	76.8 ^b^	7.2	3746 ^a,d^	150	33 ^a^	0	33.1	5.2
10	61.4 ^a,b^	10.5	3673 ^b,e^	209	35 ^a,b^	1	31.4	5.4
15	67.8	9.6	3514 ^c,f^	187	32 ^b^	3	31.1	4.6

**Table 3:** A comparison of radial stress (σr), circumferential stress (σθ), and reduced stress (σint, shrinkage stress) generated during the irradiation of experimental composites as a function of the M-POSS content (wt.%).

M-POSS Content [wt.%]	σr [MPa]	σθ [MPa]	σint [MPa]
0	8.0 ± 0.4	−9.3 ± 0.3	17.3 ± 0.8
0.5	8.2 ± 0.5	−9.5 ± 0.7	17.6 ± 1.2
2	7.1 ± 0.4	−8.3 ± 0.4	15.4 ± 0.8
10	7.2 ± 0.5	−8.5 ± 0.4	15.7 ± 0.9
15	8.8 ± 0.3	−10.1 ± 0.4	18.8 ± 0.7

**Figure A7:**
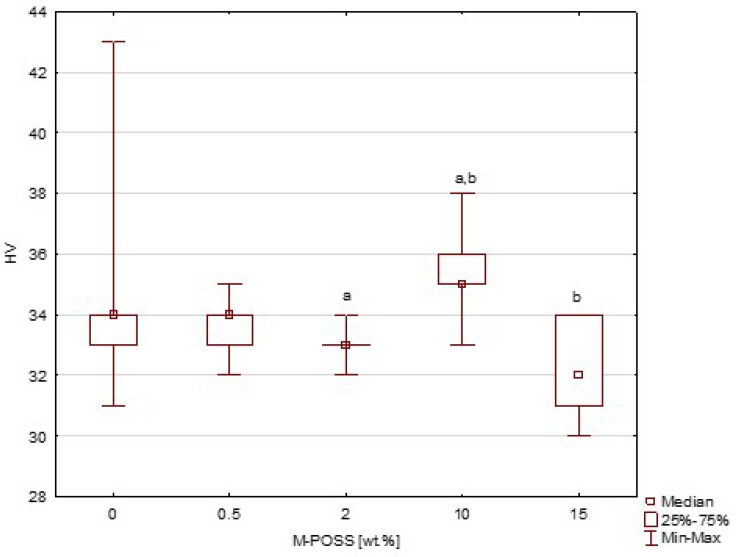
Box-and-whisker plot of Vickers hardness (HV) of tested composites with M-POSS. Statistically significant differences (the same letter) were found between the following: (a,b) 10 wt.% M-POSSS and (a) 2 wt.% M-POSS (*p*-value = 0.012760) and (b) 15 wt.% M-POSS (*p*-value = 0.002990).

## Data Availability

The original contributions presented in this study are included in the article/Supplementary Materials. Further inquiries can be directed to the corresponding authors.

## Supplementary Materials

The following supporting information can be downloaded at https://www.mdpi.com/article/10.3390/ma19061261/s1: Table S1: The raw data of mechanical properties of experimental composites.

## Appendix A

**Table A1:** The isochromatic patterns around tested composites depending on M-POSS quantity.

			
0 wt.% M-POSS	0.5 wt.% M-POSS	2 wt.% M-POSS	10 wt.% M-POSS
	
15 wt.% M-POSS			
